# Brain local structural connectomes and the subtypes of the medial temporal lobe parcellations

**DOI:** 10.3389/fnins.2025.1529123

**Published:** 2025-02-12

**Authors:** Zhensheng Li, Jie Ma, Hongmin Bai, Bingmei Deng, Jian Lin, Weimin Wang

**Affiliations:** ^1^Department of Neurology, General Hospital of Southern Theater Command, Guangzhou, China; ^2^Department of Neurosurgery, Chinese PLA General Hospital, Beijing, China; ^3^Department of Neurosurgery, General Hospital of Southern Theater Command, Guangzhou, China

**Keywords:** brain connectome, local structural connectome, medial temporal lobe, brain connectivity, local connectivity

## Abstract

**Objective:**

To investigate the quantitative characteristics and major subtypes of local structural connectomes for medial temporal lobe (MTL) parcellations.

**Methods:**

The Q-Space Diffeomorphic Reconstruction (QSDR) method was used to track white matter fibers for the ROIs within MTL based on the integrating high-resolution T1 structural MR imaging and diffusion MR imaging of 100 adult Chinese individuals. Graph theoretical analysis was employed to construct the local structural connectome models for ROIs within MTL and acquire the network parameters. These connectivity matrices of these connectomes were classified into major subtypes undergoing hierarchical clustering.

**Results:**

(1) In the local brain connectomes, the overall network features exhibited a low characteristic path length paired with moderate to high global efficiency, suggesting the effectiveness of the local brain connectome construction. The amygdala connectomes exhibited longer characteristic path length and weaker global efficiency than the ipsilateral hippocampus and parahippocampal connectomes. (2) The hubs of the amygdala connectomes were dispersed across the ventral frontal, olfactory area, limbic, parietal regions and subcortical nuclei, and the hubs the hippocampal connectomes were mainly situated within the limbic, parietal, and subcortical regions. The hubs distribution of the parahippocampal connectomes resembled the hippocampal structural connectomes, but lacking interhemispheric connections and connectivity with subcortical nuclei. (3) The subtypes of the brain local structural connectomes for each ROI were classified by hierarchical clustering, The subtypes of the bilateral amygdala connectomes were the amygdala-prefrontal connectome; the amygdala-ipsilateral or contralateral limbic connectome and the amygdala-posterior connectome. The subtypes of the bilateral hippocampal connectomes primarily included the hippocampus-ipsilateral or contralateral limbic connectome and the anterior temporal-hippocampus-ventral temporal-occipital connectome in the domain hemisphere. The subtypes of the parahippocampal connectomes exhibited resemblances to those of the hippocampus.

**Conclusion:**

We have constructed the brain local connectomes of the MTL parcellations and acquired the network parameters to delineate the hubs distribution through graph theory analysis. The connectomes can be classified into different major subtypes, which were closely related to the functional connectivity.

## Introduction

The medial temporal lobe (MTL) is one of the key functional centers in the human brain, comprising the amygdala, hippocampus, and parahippocampal cortex. An evolutionary perspective has revealed the significance of MTL development in humans, emphasizing its status as a hallmark trait that distinguishes human from that of other primates in the neuroanatomy viewpoint (Vos de Wael et al., 2021). These intricate structures form extensive neural connections with diverse cortical regions, thereby facilitating the coordination of various higher-order cognitive functions. Numerous studies have highlighted the essential role of MTL in shaping memory encoding, semantic processing, and emotional regulation (Vaz et al., 2019; Phelps, 2004; Ralph et al., 2017). Despite significant advancements, the complete picture of MTL functionality remains to be fully understood. Ongoing neuroscientific research has steadily uncovered its involvement in additional aspects of human cognition, including computational processes and visuospatial perception (Lisman et al., 2017; Zhang et al., 2023).

Currently, researchers are actively investigating brain network models derived from the attributes of the whole-brain connectivity matrix through graph theoretical analysis (Rolls et al., 2023a, 2022a; Luo et al., 2024; Indovina et al., 2020). However, it is important to note that connections between brain regions are not uniformly distributed across the entire brain; instead, local connections formed between specific regions are closely tied to particular functions. For instance, the connections between the medial temporal lobe structures and various cortical areas are intricately linked to distinct functions: emotional processing is closely associated with the amygdala, prefrontal cortex, and cingulate gyrus, while memory functions are reflected in the relationships between the hippocampus and other regions of the limbic system. Moreover, disruptions in these specific connections, whether structural or functional, can result in significant neurological disorders. For example, structural deterioration of the hippocampal-limbic network is a pathological hallmark of Alzheimer’s disease, while functional abnormalities in the MTL can lead to conditions such as MTL epilepsy and schizophrenia (Lieberman et al., 2018; Bernhardt et al., 2016; Tetreault et al., 2020). Therefore, models of the brain’s local connectome hold substantial promise for elucidating their functional roles, analyzing the underlying network mechanisms of clinical neurological symptoms, and identifying modifiable hubs within brain-machine interfaces. Additionally, there remains a notable gap in the quantitative analysis of local network parameters, as well as a deficiency in the subtyping of brain connectivity matrices.

We hypothesized that the local connectomes of the MTL parcellations could be elucidated and quantified through the advancement of diffusion magnetic resonance imaging (dMRI). Initially, we reconstructed the white matter tracts associated with regions of interest (ROIs) in the MTL using Q-Space Diffeomorphic Reconstruction (QSDR), which integrated diffusion MRI (dMRI) data with high-resolution T1-weighted imaging. Subsequently, we employed graph theoretical analysis to construct local structural connectome models for these ROIs within the MTL and to derive the associated network parameters. The connectivity matrices obtained from these models underwent hierarchical clustering, thereby laying a foundation for a comprehensive investigation of structural connectivity and paving the way for functional studies centered on the MTL structures.

## Methods

### Participants

This study included a sample of 100 healthy adult Chinese individuals, comprising 50 males and 50 females, with ages ranging from 20 to 38 years (mean age 27.71 ± 4.00 years). All participants were right-handed, had attained a bachelor’s degree or higher, and had no history of neurological disorders, mental illnesses, or chronic diseases, such as hypertension, diabetes, or coronary heart disease. Written informed consents were obtained from all participants and their legal guardians. To ensure cognitive and neurological normality, all participants underwent the intelligence quotient test base on the Wechsler Adult Intelligence Scale III Chinese revised version (WAIS III-RC, copyright by Human Map Publishing Company, ISBN 50132001), electroencephalography examination, and brain MRI scan. All the results were normal. A two-sample two-tailed *t*-test was conducted, which demonstrated no significant statistical difference between males and females in terms of age and scores of the WAIS III-RC (Table 1).

**Table 1:** The ages and scores of the WAIS III-RC of the healthy adult Chinese individuals.

	Male (50)	Female (50)	*t*	*p*
Ages (years)	26.93 ± 5.47	27.73 ± 6.23	−0.881	0.38
Verbal intelligence quotient	115.72 ± 4.26	115.08 ± 4.11	0.764	0.447
Performance intelligence quotient	116.66 ± 3.76	116.08 ± 2.96	0.857	0.394
Total intelligence quotient	116.16 ± 2.74	115.64 ± 2.50	0.992	0.324

### Methods of MRI scanning

The 3D T1-weighted structural MRI was performed using a Siemens Skyra 3 T MRI scanner. Magnetization-Prepared Rapid Gradient Echo (MPRAGE) sequence was employed to acquire the images, with the following parameters: TE = 3.24 ms, TR = 2,300 ms, TI = 900 ms, flip angle = 9 degrees, band width = 210 Hz/pixel, FOV = 256 × 256, and a resolution of 1.0 × 1.0 × 1.0 mm^3^, resulting in a total of 240 sagittal slices.

The diffusion MR Imaging (dMRI) was performed using a Spin-echo EPI sequence to obtain diffusion-weighted images in 65 gradient directions. The diffusion weighting consisted of two shells with b values of 0 and 1,000/mm^2^. The diffusion directions were uniformly distributed in the q-space shells and were optimized such that each contiguous directional subset was isotropic. The acquisition parameters were as follows: TR = 10,000 ms, TE = 90 ms, FOV = 256 × 256, slice thickness = 2 mm, with 75 slices and 2 mm isotropic voxels. The echo spacing was 0.75 ms, the bandwidth was 1,502 Hz/Px, and the phase partial Fourier was 6/8. A complete diffusion MRI run took approximately 11 min and 22 s.

### Methods of MRI data processing

Integration of dMRI and MPRAGE sequence: The DSI Studio software package was utilized to process the dMRI data. Initially, the dMRI data were converted into a 4D-NIFTI file, along with the b-value and localization files (bval, bvec files), which were then imported into the DSI Studio software to generate multiple SRC files. Subsequently, the SRC files were co-registered with MPRAGE data to the MNI space to facilitate the integration of imaging modalities. The QSDR was employed for fiber tracking with a diffusion sampling length ratio of 1.25, utilizing the ICBM152_adult template. This method is the MNI adaptation of Generalized Q-sampling Imaging (GQI) reconstruction approach. It can calculate the orientation distribution function (ODF) of water diffusion using Fourier transforms and numerical integration in MNI space, providing a direct analytical relationship for the diffusion ODF to avoid errors in numerical estimation. To ensure data accuracy, the Check b-table was used to verify and rectify any flips or exchanges in the b-table in the x-, y-, or z-directions, enabling the generation of the FIB file after reconstruction.

The FIB file was selected for white matter fiber tractography, utilizing Anatomical Automatic Labeling 2 (AAL2) template partitions. The regions of interest (ROIs) including the amygdala, hippocampus, and parahippocampal gyrus within MTL, were chosen for probability tracking. Tracking parameter was set as the qa value, with a tracking threshold of 0.2, an angle threshold of 30°, and tracking length ranging from 30 mm to 200 mm, and 10,000 seeds were employed to tract the white matter fiber model for the ROIs within MTL.

### Statistical analysis

The graph theoretical analysis module of DSI Studio was employed to construct the brain local connectome models for the ROIs in MTL and to compute the global complex network topology parameters including the characteristic path length and global efficiency. Additionally, parameters of the brain local connectomes for ROIs were extracted. These parameters included the degree, connectivity strength, local efficiency, clustering coefficient (Bullmore and Sporns, 2009).

#### Characteristic path length

It refers to the average of all shortest path lengths between all pairs of nodes (*n*) in the network. A smaller characteristic path length indicates a faster information transmission speed within the network. To express the characteristic path length, the sum of the shortest distances (Dis) between all nodes (*i*, *j*) in the network is first calculated to obtain the total path length *CP_T_*, and the characteristic path length *C_T_* is defined as the average of *CP_T_*.


$$CP_{T}=\sum Dis\left(i,j\right),CP=CP_{T}/\left\{n\left(n-1\right)\right\}$$

Where *n* is the total number of nodes in the network.

#### Global efficiency

It is a measure of parallel information transmission in the given network, defined as the inverse of the sum of all shortest path lengths in this network.


$$GE=\left\{\sum 1/CP\left(i,j\right)\right\}/\left\{n\left(n-1\right)\right\}$$

#### Degree

It refers to the number of neighboring nodes that are directly connected to a given node within the network. Degree is a metric used to evaluate the breadth of a given node’s connections; nodes with a higher degree have more extensive connectivity within the network.


$$D_{i}=\sum C\left(i,j\right)$$

where *C*(*i*,*j*) represents the connection status between nodes *i* and *j*, with a connection = 1 and no connection = 0.

#### Strength

It refers to the cumulative sum of the weights of connections between a specific node and its neighboring nodes within the network.


$$S_{i}=\sum W\left(i,j\right)$$

where *W*(*i*,*j*) represents the connection weight between nodes *i* and *j*.

#### Clustering coefficient

It is the ratio of the actual edges between all neighboring nodes (*n*) connected to node i and the maximal possible edges between these neighboring nodes. It primarily reflects the cohesiveness and stability of connections for a given node within the network.


$$CC_{i}=2R_{i}/\left\{D_{i}\left(D_{i}-1\right)\right\}$$

where *R_i_* is the number of neighboring nodes connected to node *i*.

*n_i_* is the degree of node i, representing the number of neighboring nodes connected to node *i*.

The algorithms for local efficiency and global efficiency are similar. However, local efficiency is computed at the level of individual nodes rather than at the level of the global network.

In addition, we extracted the qa values in the AAL2 parcellations from the white matter fiber models for each ROI. The qa value is an indicator of diffusional anisotropy in biological tissues and provides a more precise reflection of realistic white matter fibers compared to the traditional fa values from DTI method, as it can better conform to the rules of H_2_O molecule flow (Yeh et al., 2013). The connectivity matrix was computed by the coherence analysis of the qa values in the AAL2 parcellations. All the participants’ connectivity matrices of white matter fiber models for each ROI were averaged and normalized, with average coherence values less than 0.2, ignored.

Statistical analysis was performed on both the participants’ general information data and the normalized and averaged connectivity matrices using the Origin2022b software package. The chi-square test was used to compare categorical data, whereas the analysis of variance (ANOVA) was conducted for the comparisons of network parameters, and Fisher’s Least Significant Difference (LSD) for post-hoc statistical test was used to identify where significant differences exist between group means after ANOVA.

**Figure 1:**
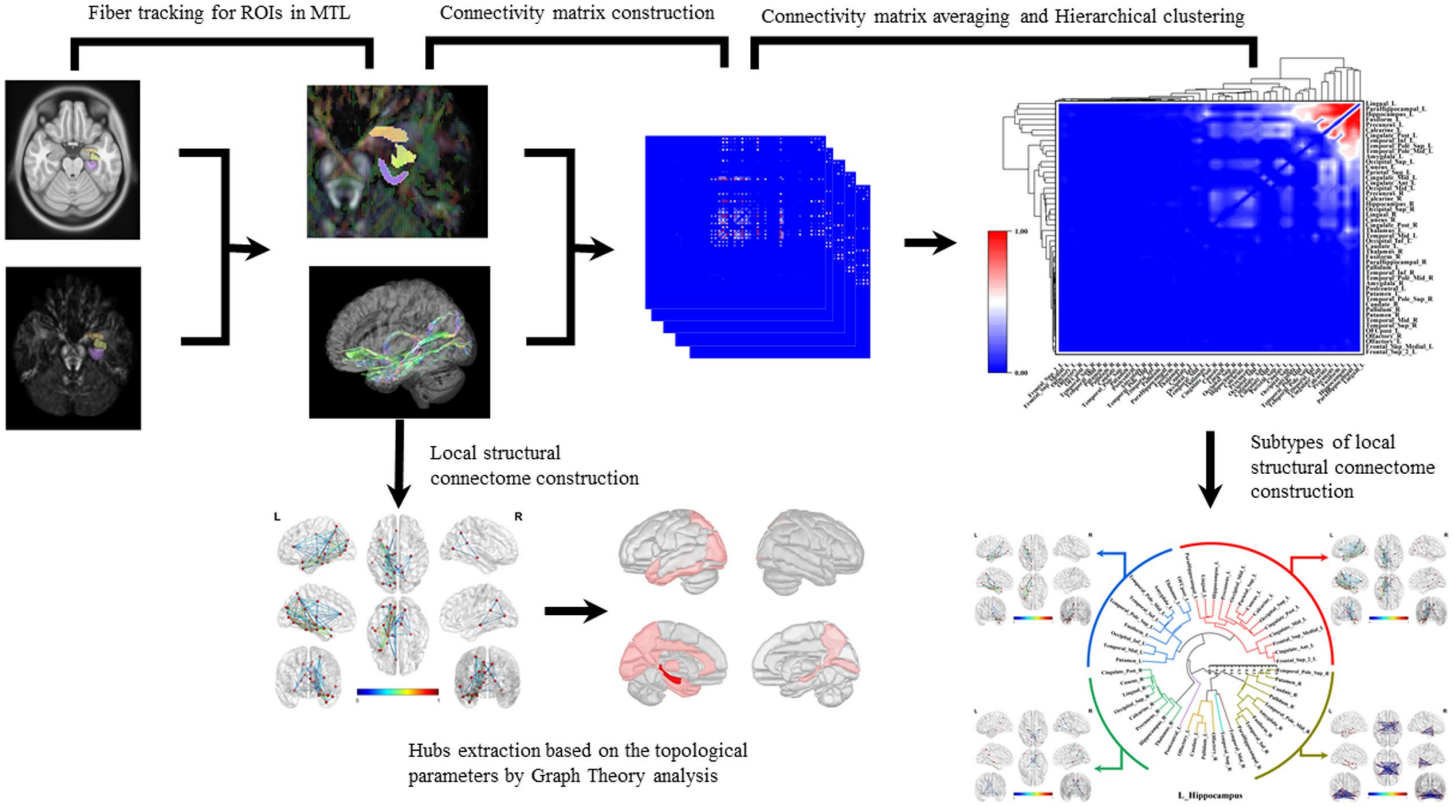
Construction of brain local structural connectome and hierarchical clustering. Diffusion MRI was co-registered with MPRAGE sequence images to the MNI space. The subregions within MTL were selected as ROIs, and the fiber probability tracking was performed with tracking parameters setting to qa value. Graph theoretic analysis was used to calculate the global and local network topological parameters, and the nodes with top one-third of local network parameters were identified as hubs in these structural connectomes. The connectivity matrix was computed by analyzing the coherence of the qa values in the AAL2 parcellations. The connectivity matrices of all the white matter fiber models for each ROI were averaged and normalized. The hierarchical cluster analysis was applied to the connectivity matrices to acquire the brain connectome subtypes.

Finally, the statistical method of hierarchical clustering was applied to the connectivity matrices to categorize the subtypes of brain local structural connectomes. Hierarchical clustering is a widely utilized technique for cluster analysis. It merges a new cluster based on the distance or similarity between samples or groups. This method can be categorized into inter-sample and inter-group distance approaches. In this study, we adopt of inter-group distance methodology, distinct group. We implemented the average group linkage algorithm to merge groups that are close in average distance into clusters: *D(A,B) = {(∑a∈A) × (∑b∈B) × d(a,b)} /(/A/×/B/)*, where *A* and *B* are the respective groups and *d(a,b)* represents the distance between individual elements (Figure 1).

## Results

### Establishment of the brain local structural connectomes for each ROI within MTL

**Figure 2:**
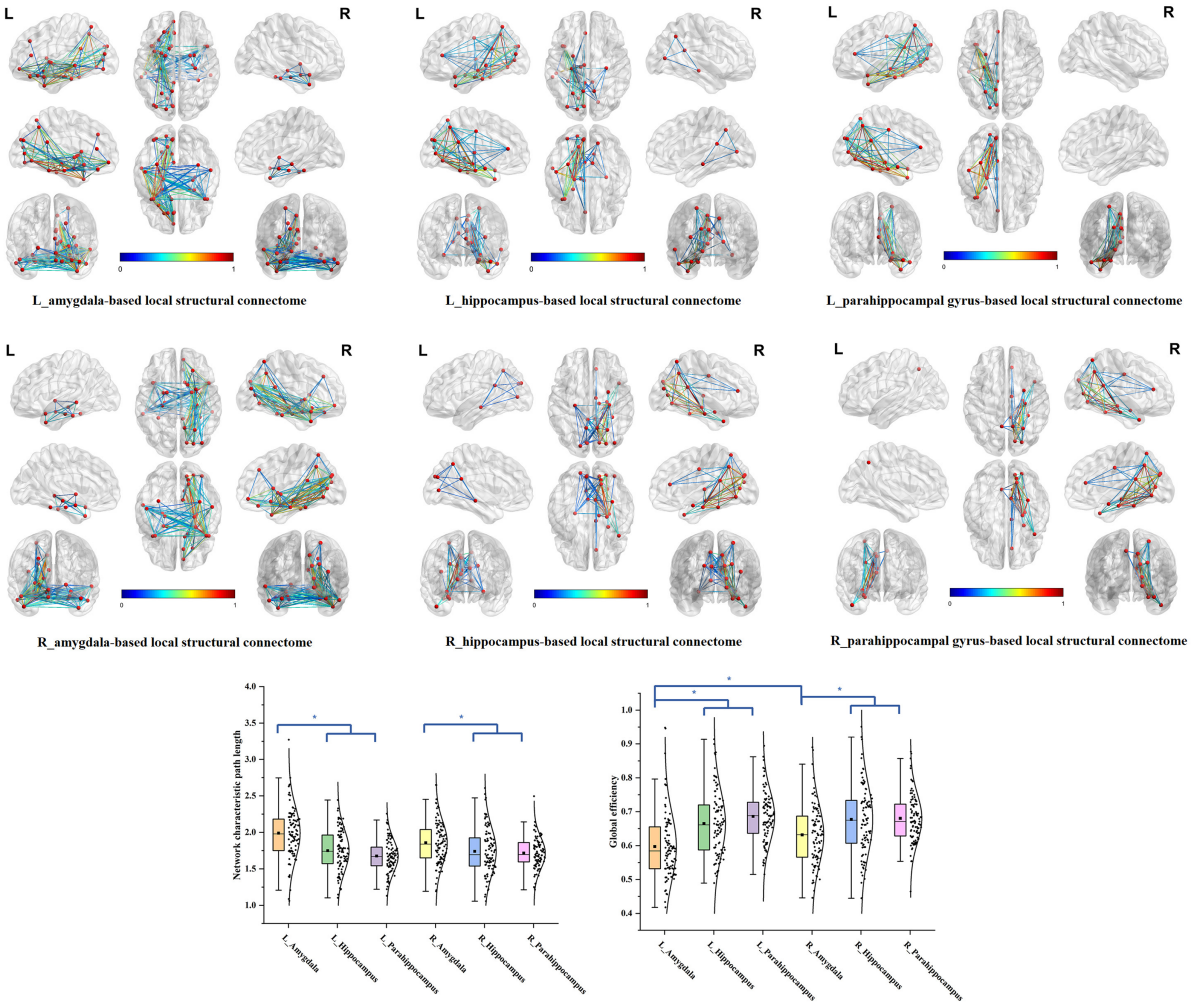
The brain local structural connectomes for each ROI and the comparison of the network global topography parameters between the MTL parcellations. The brain local structural connectomes for each ROI, including the amygdala, hippocampus, and the parahippocampal gyrus, were delineated. The ranges of the normal values for the global topology parameters CP and GE were derived by graph theory analysis. The bilateral amygdala structural connectomes exhibited significantly longer CP and weaker GE, compared to the ipsilateral hippocampal and parahippocampal connectomes. Furthermore, the GE of the left amygdala structural connectome model was lower than that of the right amygdala.

We delineated the local white matter fiber models for each ROI, including the amygdala, hippocampus, and the parahippocampal gyrus. By employing graph theoretical analysis, we successfully established all the brain local structural connectomes for the ROIs (Figure 2). Within these connectomes, the normal value range of the global network topology parameters can be derived, that exhibited the characteristic with a low characteristic path length paired with moderate to high global efficiency (Table 2). Our findings also demonstrated that, compared to the ipsilateral hippocampal and parahippocampal connectome, the amygdala connectome exhibited significantly longer CP and weaker GE. Furthermore, the GE of left amygdala connectome was lower than that of right amygdala.

**Table 2:** Global parameters of local structural connectome for the ROIs in MTL.

	Global efficiency	Characteristic path length
	*χ* ± Std	*χ* ± Std
L_Amygdala	0.597 ± 0.099	1.990 ± 0.357
L_Hippocampal	0.665 ± 0.096	1.749 ± 0.289
L_Parahippocampal	0.686 ± 0.077	1.676 ± 0.225
R_Amygdala	0.632 ± 0.089	1.857 ± 0.292
R_Hippocampal	0.677 ± 0.102	1.741 ± 0.325
R_Parahippocampal	0.680 ± 0.072	1.716 ± 0.219

Within these connectomes, there were certain nodes that establish numerous and stable connectivity with surrounding nodes, which are referred to as hubs. These hubs typically exhibited high local network parameters, such as elevated degree, strong connectivity strength, high clustering coefficient and local efficiency. By extracting the average parameter values from the local structural connectomes for each ROI, we identified the top one-third hubs in these structural connectomes.

**Figure 3:**
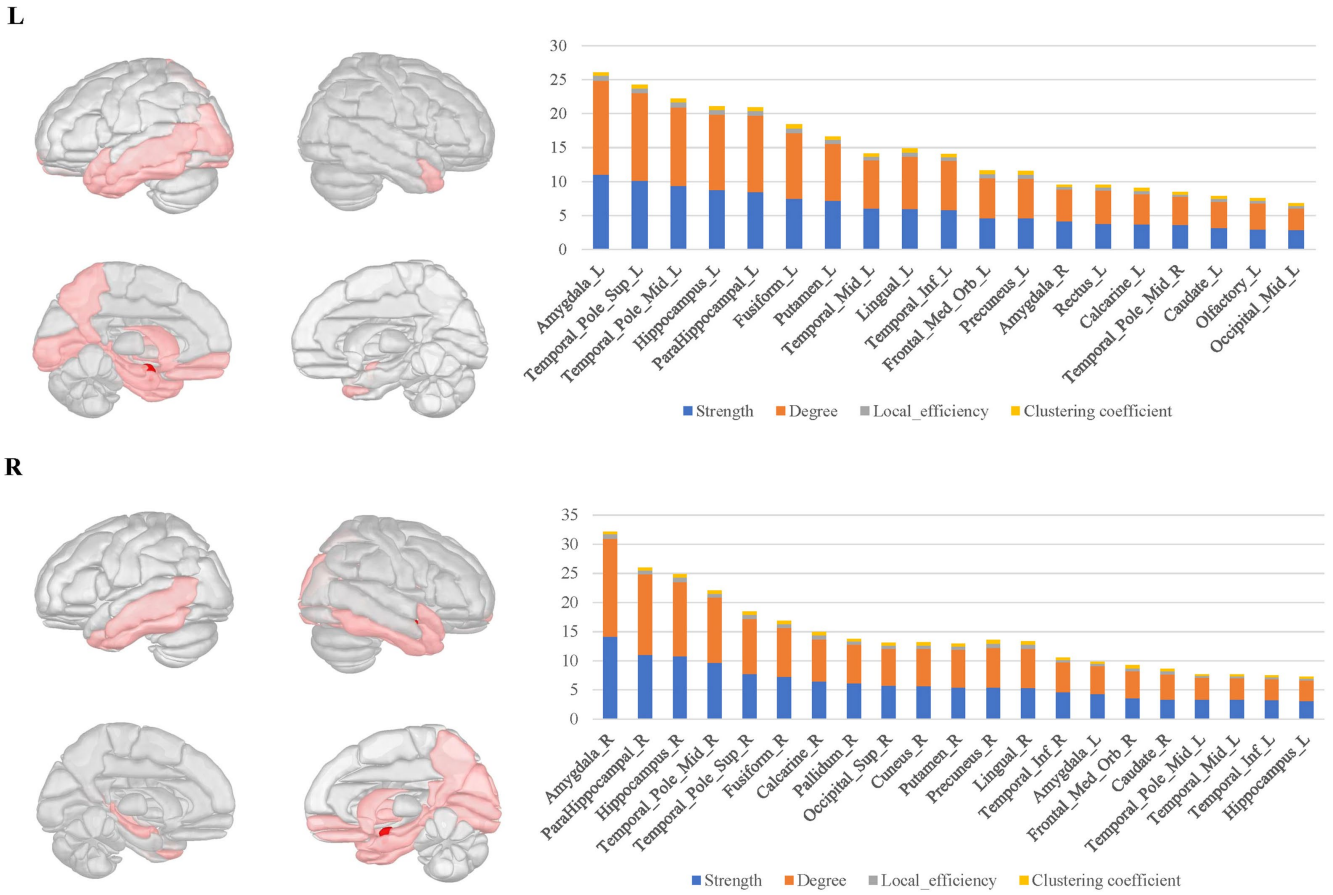
The hubs distribution of the bilateral amygdala connectomes. The hubs of the bilateral amygdala connectomes were distributed in the ventrolateral prefrontal cortex, olfactory area, limbic regions, posterior cingulate cortex, and subcortical nuclei in the bilateral amygdala local structural connectomes. There were also connections between the bilateral amygdala regions.

In the bilateral amygdala connectomes, the hubs were distributed in the ventrolateral prefrontal cortex, olfactory area, limbic regions, medial parietal and occipital cortex, and subcortical nuclei. The ventrolateral prefrontal cortex included the anterior and medial parts of orbitofrontal gyrus, rectus gyrus and the limbic regions included other structures than the amygdala within MTL, such as the hippocampus, parahippocampal gyrus, anterior cingulate gyrus, anterior and ventral temporal lobe cortex. Additionally, the hubs included certain subcortical nuclei, such as the caudate nucleus and globus pallidus. There were also connections between the bilateral amygdala (Figure 3).

**Figure 4:**
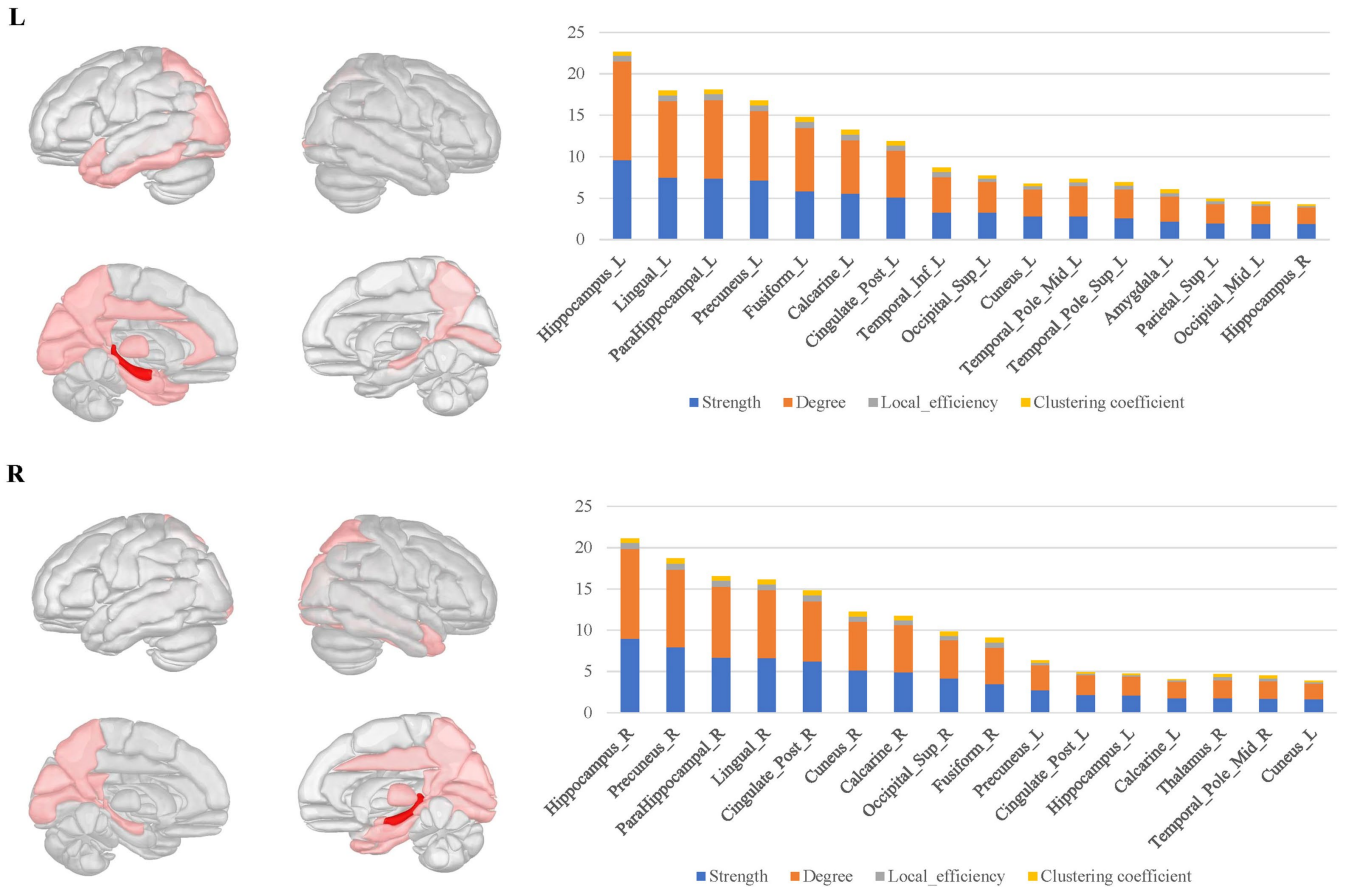
The hubs distribution of the bilateral hippocampal connectomes. For the bilateral hippocampal local structural connectomes, the hubs were primarily located in the limbic regions, thalamus, and posterior cerebral cortex. Important connections between bilateral hippocampus were also observed.

For the bilateral hippocampal connectomes, the hubs were primarily located in the limbic regions, thalamus, and posterior cerebral cortex. In addition to the regions shared with the amygdala connectome in the limbic regions, there were also connections to other limbic regions through the fornix fibers, such as the middle and posterior cingulate gyrus. Moreover, the medial parietal and occipital regions were involved as well. Important connections between bilateral hippocampus were also observed (Figure 4).

**Figure 5:**
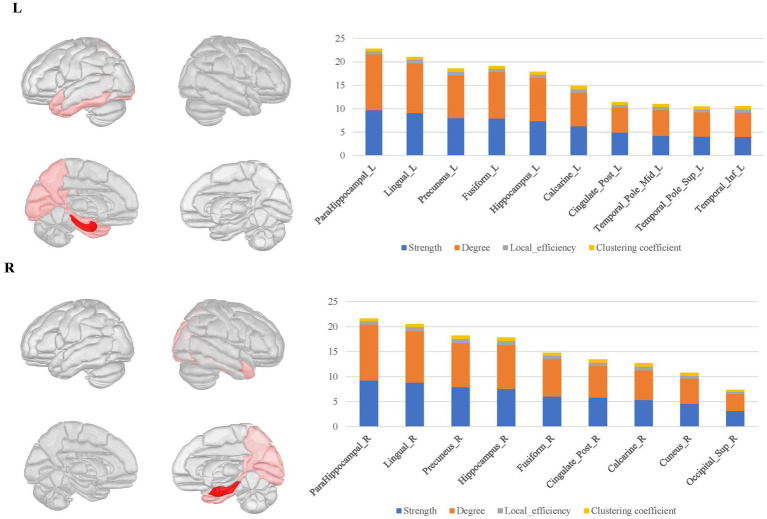
The hubs distribution of the bilateral parahippocampal connectomes. The bilateral parahippocampal local structural connectomes showed a certain degree of similarity to that for the hippocampus, with hubs mostly concentrated in the limbic regions and the medial surface regions of the parietal and occipital lobes, but lacking interhemispheric connections and connectivity with subcortical nuclei.

The bilateral parahippocampal connectomes showed a certain degree of similarity to that of the hippocampus, with hubs mostly concentrated in the limbic regions and the medial parietal and occipital regions, but corresponding to fewer hubs relative to the hippocampal connectomes, as well as lacking the interhemispheric connections and the connectivity with subcortical nuclei (Figure 5).

### Cluster analysis of the local structural connectomes for each ROI within MTL

In this section, we demonstrated the hierarchical clustering analysis of the normalized average connectivity matrices for each ROI to categorize the subtypes of local structural connectomes within the MTL structures.

**Figure 6:**
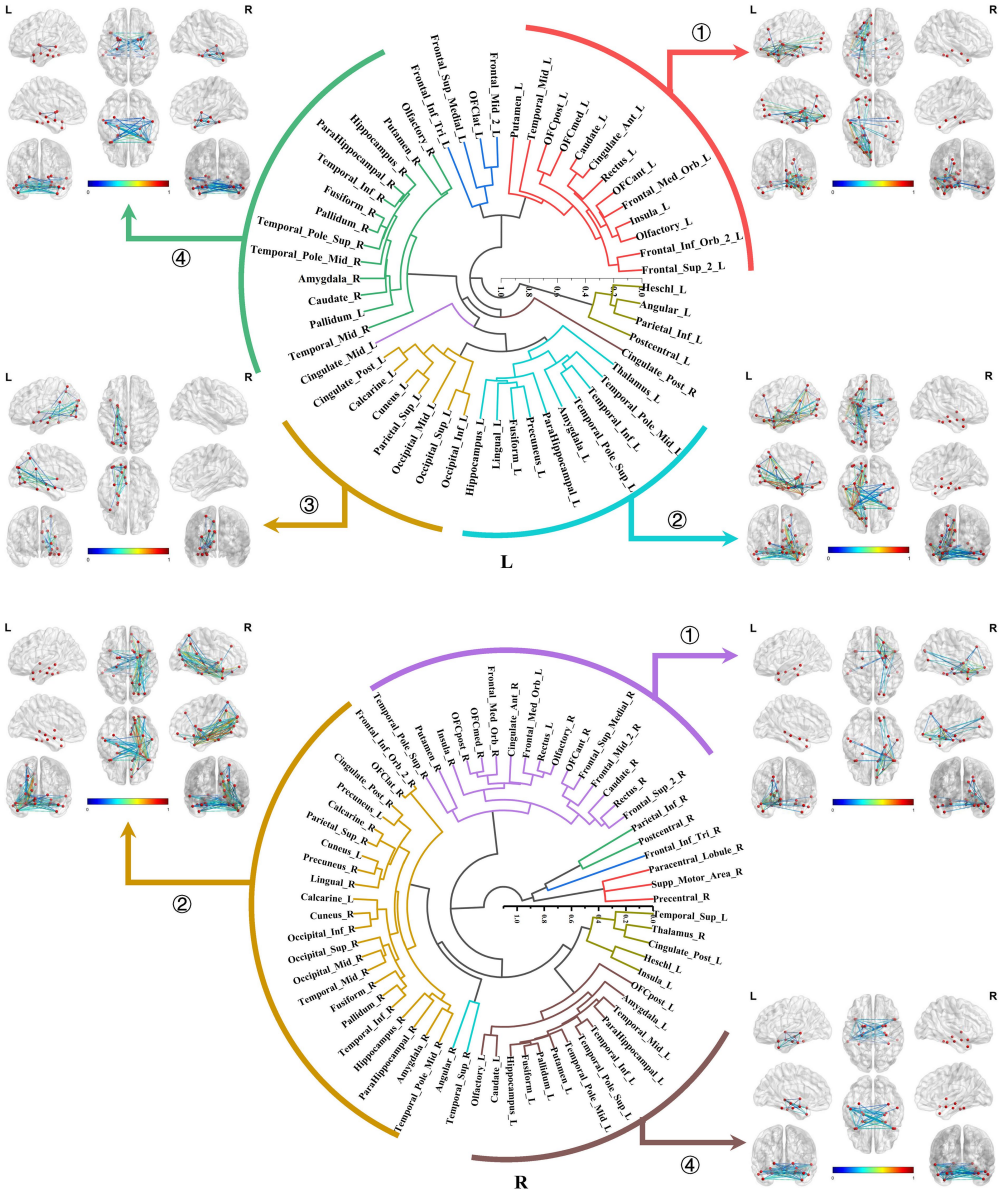
Hierarchical clustering of the bilateral amygdala connectomes. The primary subtypes presented in the bilateral amygdala connectomes included: ① The amygdala-prefrontal connectome; ② The amygdala-limbic connectome; ③ The amygdala-posterior connectome; ④ The amygdala-contralateral limbic connectome.

The primary subtypes presented in the bilateral amygdala connectomes were similar and included the following: ① The amygdala-prefrontal connectome, involved regions such as the orbitofrontal gyrus, anterior cingulate gyrus, insula, rectus gyrus, olfactory area, and portions of the lateral frontal lobe connected to the amygdala. ② The amygdala-limbic connectome, encompassed other structures in the medial and ventral temporal lobes, thalamus, and parts of the subcortical nuclei connected to the amygdala. ③ The amygdala-posterior connectome, primarily consisted of extensive connections between the amygdala and the parietal, occipital lobes. In the right hemisphere, the amygdala–posterior connectome potentially merged with the amygdala–limbic connectome. ④ The amygdala-contralateral limbic connectome, mainly involved connections between the amygdala and contralateral limbic structures and subcortical nuclei (Figure 6).

**Figure 7:**
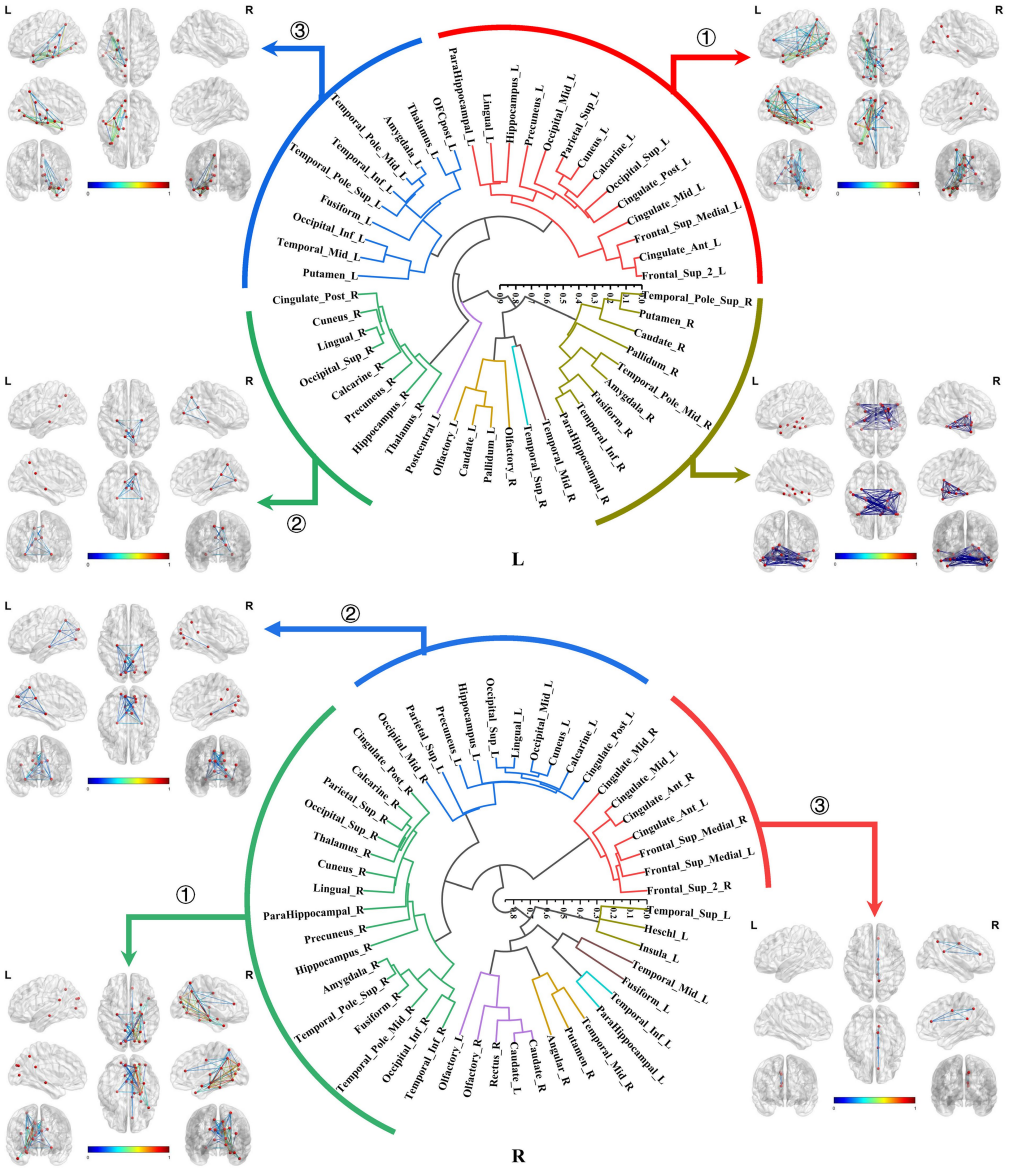
Hierarchical clustering of the bilateral hippocampal connectomes. Both the bilateral hippocampal connectomes exhibited three primary subtypes respectively, with two showing consistency: ① The hippocampus-limbic connectome; ② The hippocampus-contralateral limbic connectome; ③ The third subtype of the left hippocampal local structural connectome was the anterior temporal-hippocampal temporal-occipital connectome, while the third subtype of the right hippocampal local structural connectome was the hippocampus–cingulate–medial frontal connectome.

Both the bilateral hippocampal connectomes exhibited three primary subtypes respectively, with two showing consistency: ① The hippocampus-limbic connectome, primarily comprised connections between the hippocampal region and other ipsilateral structures within MTL, posterior cingulate gyrus, and parts of the medial parietal and occipital regions. ② The hippocampus-contralateral limbic connectome, resembled the amygdala-contralateral limbic connectome and involved connections with the contralateral hippocampus, thalamus, posterior cingulate gyrus, and portions of the medial parietal and occipital regions. ③ The third primary subtype of the left hippocampal connectome was the anterior temporal-hippocampal-ventral temporal-occipital connectome, which was primarily related to connections between the anterior temporal lobe, inferior temporal lobe, and inferior occipital lobe associated with the hippocampus. Conversely, the third primary subtype of the right hippocampal connectome was the hippocampus–cingulate–medial frontal connectome, which involved the ipsilateral cortical regions from the anterior and middle cingulate gyri to the medial frontal lobe connected to the right hippocampus (Figure 7).

**Figure 8:**
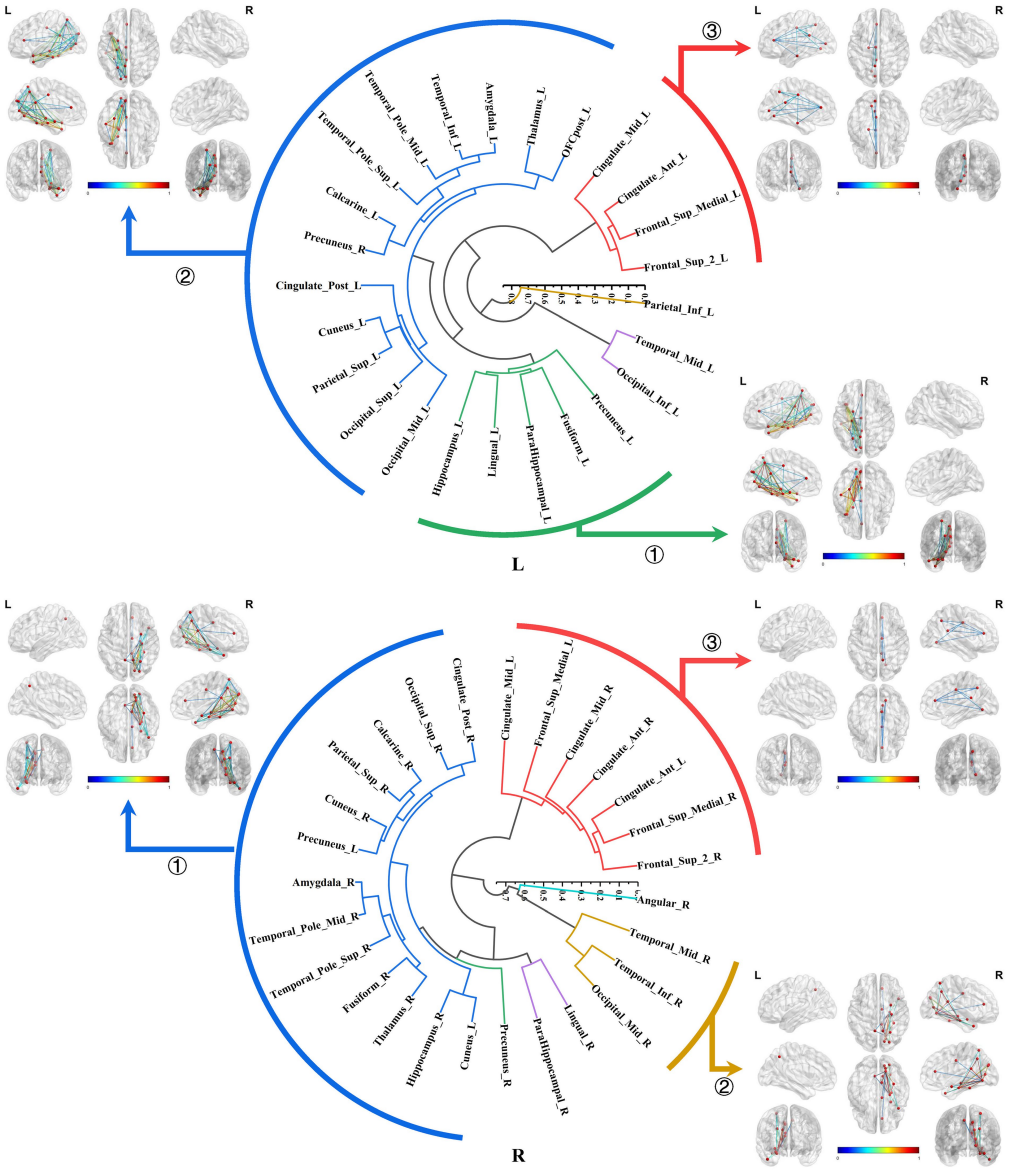
Hierarchical clustering of the bilateral parahippocampal connectomes. The bilateral parahippocampal connectomes could also be classified into three primary subtypes, exhibiting a certain degree of similarity in subtypes of the hippocampal connectomes: ① The parahippocampal-limbic connectome; ② The parahippocampal-ventral temporal-occipital connectome; ③ Continuous connection from the cingulate gyrus to the medial frontal lobe, which was associated with the ipsilateral parahippocampal gyrus.

The bilateral parahippocampal connectomes could also be classified into three major subtypes, exhibiting a certain degree of similarity in subtypes of the hippocampal connectomes: ① The parahippocampal-limbic connectome, included other structures within the MTL, thalamus, cingulate gyrus, and the medial surface of the parietal and occipital cortex associated with the ipsilateral parahippocampal gyrus. ② The parahippocampal-ventral temporal-occipital connectome, contained the ipsilateral cortex of the ventral temporal and occipital lobes connected to the parahippocampal gyrus. ③ The third major subtype of the parahippocampal structural connectome indicated a continuous connection from the cingulate gyrus to the medial frontal lobe, which was associated with the ipsilateral parahippocampal gyrus, a subtype similar to the third subtype of the right hippocampal connectome (Figure 8).

## Discussion

In the domain of neuroscience, there is a growing consensus that the functions are structurally and functionally integrated by specific brain regions (Axer and Amunts, 2022; Zhang et al., 2023; Tremblay and Dick, 2016), while the white matter fibers play a crucial role in the process. Traditional neuroanatomy has been a vital method for studying white matter tracts, however it has limitations such as the inability to perform *in vivo* studies and the lack of quantitative processing capabilities. Diffusion MRI (dMRI) can effectively address these limitations (Yeh et al., 2018). In recent years, researchers have advocated the development of novel methods to analyze the HCP data and discover brain connectomes closely associated with brain function (Jbabdi et al., 2015; Thiebaut de Schotten and Forkel, 2022). Nevertheless, there are inherent structural differences in the brains of individuals from different geographical regions and cultures (Ge et al., 2023; Tooley et al., 2021). Furthermore, specific databases for the Chinese population are lacking. Therefore, a multiple model imaging database of Chinese individuals was collected and used in this study.

In clinical practice, there is a greater emphasis on the localization and intervention of brain connectomes that associated with certain neurological functions and symptoms. These connectomes are predominantly situated within the connections between the local functional areas and specific regions (Laberge et al., 2006; Gothard, 2020). Uncovering these local connectomes has greater clinical advantages than that of whole-brain connectomes. Therefore, we proposed the concept of brain local connectome in this study, defined as connectomes formed by tracking specific regions connected to local cerebral regions and conducted the quantitative analysis. Initially, we selected functional subregions within MTL as the ROIs to track the local white matter fibers. Subsequently, we applied a graph theoretical analysis to establish and quantitatively analyze the brain local connectomes.

In the local brain connectomes, the overall network features exhibited a low characteristic path length paired with moderate to high global efficiency, suggesting the effectiveness of the local brain connectome construction. In the amygdala connectome, hubs were located in regions including the ventral prefrontal cortex, olfactory area, limbic system, parietal-occipital cortex, and subcortical nuclei. Notably, these hubs exhibited extensive connectivity, even establishing links with posterior regions of the brain. This extensive connectivity may account for the longer characteristic path length compared to the hippocampal and parahippocampal connectomes, which displayed correspondingly lower global efficiency. In contrast, the hubs in the hippocampal and parahippocampal connectomes were mainly situated in the limbic regions and parietal-occipital cortex. In addition, connections between the interhemispheric amygdala and hippocampus were observed in the corresponding connectomes. The distribution of these hubs was closely linked to the functional integration of the local connectomes for the ROI. The fMRI results have revealed effective functional connectivity of the MTL (Paquola et al., 2020; Hung et al., 2020; Chen et al., 2020; Seoane et al., 2022), aligned with the local structural connectomes for the subregions within MTL in this study, corroborating the unity of structure and function in the local connectomes of MTL.

In the second section, we conducted hierarchical clustering of the brain local structural connectomes for each ROI to identify the subtypes. Current perspectives suggested that the brain functional diversity was mediated by the formation of distinct network modules. Hierarchical clustering, a tree-based method that organized data based on inter-group similarity, has been employed to group brain regions according to the similarity of their connectivity features. The regions that formed these clusters were considered integral components of brain networks, and this approach has increasingly been applied in research on brain network detection. Early studies utilized hierarchical clustering on electroencephalographic signals at different frequencies to reveal intrinsic brain connectivity networks, thereby enhancing our understanding of how the brain regulated cognitive processes (Ozdemir et al., 2015; Park et al., 2019). In recent years, researchers have combined dictionary learning algorithms with hierarchical clustering to extract stable, macro-scale brain network interactions from task-based fMRI data in the Human Connectome Project (Yuan et al., 2022). This provided compelling evidence for the utility of hierarchical clustering in brain connectome analysis.

We identified four main subtypes for the bilateral amygdala connectomes. As an integral component of the limbic regions, the amygdala maintains close connections with other MTL structures, primarily facilitating emotional and memory function. Notably, the amygdala plays a prominent role in emotional regulation, forming emotion modulation networks predominantly through its connections with the ventral prefrontal cortex and anterior cingulate cortex (Wang et al., 2022; Underwood et al., 2021), corresponding to the subtype of amygdala–prefrontal connectome identified in our study. Such connections have also been observed in primates, representing a crucial evolutionary feature of these species (Laberge et al., 2006). Several researches have revealed that amygdala-prefrontal coupling activation is implicated in the memory consolidation pathways of certain negative emotions (e.g., fear and anxiety), thereby enhancing vigilance and defense against potential threats in real-world scenarios. This functional strengthening of the amygdala–prefrontal cortex may be the mechanism underlying various psychiatric and affective disorders (Gothard, 2020; Moscarello and Penzo, 2022; Sladky et al., 2024). Moreover, amygdala enlargement is usually exhibited in patients with panic seizures, and the electroencephalogram during seizures shows epileptiform discharges propagating from the MTL to the prefrontal cortex (Chen et al., 2021; Jha et al., 2023; Méndez-Bértolo et al., 2016).

Additionally, it could be found connections existing between the amygdala and parietal-occipital lobes, encompassing the lateral and medial parietal cortices and visual cortical areas. In healthy individuals, primary sensory information from the parietal-occipital regions can activate the amygdala-prefrontal and amygdala-orbitofrontal circuits in response to threat or reward stimuli, enabling subjects to make appropriate behavioral decisions (Morrow et al., 2020; Gangopadhyay et al., 2021; Wassum, 2022).

The hippocampus, a core structure within the MTL, has been extensively studied in the context of brain connectivity. Building upon previous research, this study further categorized the bilateral hippocampal connectomes, revealing three significant subtypes, respectively. The hippocampus exhibited intricate connections with the limbic regions, with the pathway extending through the posterior cingulate cortex and the medial parieto-occipital cortex, progressively radiating towards the mid-cingulate cortex and portions of the medial frontal cortex. Previous research has demarcated the hippocampal memory function could be divided into ventral and dorsal streams, colloquially known as the “what” and “where” information streams, representing hippocampal pathways for episodic memory regarding the occurrence and location of events (Rolls et al., 2022a; Ma et al., 2022; Huang et al., 2021; Sormaz et al., 2017).

Our findings elucidated the hippocampus-limbic connectome as the primary pathway encompassing the dorsal “where” information stream. The key aspects of effective connectivity in the “where” stream prominently feature functional links between the hippocampus and the middle, posterior cingulate cortex, retrosplenial cortex, and medial parieto-occipital cortex, consistent with the first subtype of the local structural connectome in this study.

This connectivity is essential for providing spatial information for episodic memory, with attenuations in this connectivity leading to amnesia (Rolls et al., 2023a, 2023b, 2023c). In patients with memory-impairing conditions, such as medial temporal lobe epilepsy and Alzheimer’s disease, cortical atrophy and tau protein deposition were also evident in the connected limbic regions other than MTL (Franzmeier et al., 2019; Tavakol et al., 2019; Tetreault et al., 2020; Park et al., 2022). Our previous studies corroborated these findings, demonstrating that the cortical atrophy distributions in patients with medial temporal lobe epilepsy correlated with the spread of abnormal discharges and that the patterns of cortical atrophy were correlating with the memory function (Li et al., 2021; Li et al., 2022; Li et al., 2023).

In contrast, the anterior temporal-hippocampus-ventral temporal-occipital connectome is identified as a critical pathway for substantial connectivity of the hippocampus in the dominant hemisphere. This was also uniquely observed in the subtype of left hippocampal connectome in this study, which represented the ventral “what” information stream. This is likely due to its association with functional specialization of the dominant hemisphere in semantic memory processing (Rolls et al., 2022b; Rahimi et al., 2022; Hung et al., 2020). Previous investigations have corroborated the notion that anterior temporal structures participated in the processing of semantic memory information. Additionally, in clinic, the anterior temporal cortical atrophy, as well as their connectivity disturbances have been observed in patients afflicted with primary progressive aphasia (Battistella et al., 2019). Semantic processing necessitates an interconnected network involving the structures encompassing the inferior longitudinal fasciculus, which was coincident with the pathway identified in the third subtype of the left hippocampal connectomes in our study. Also, this pathway also implicated the critical role of hippocampus in lexical memory retrieval (Sierpowska et al., 2019; Agosta et al., 2013).

The subtypes of the bilateral parahippocampal connectomes were similar to those of the bilateral hippocampus. Our investigations have likewise uncovered that the subtypes were primarily defined by connectivity patterns between the parahippocampal gyrus and the limbic system, as well as the ventral temporo-occipital cortex, diverging from the hippocampal connectomes in the absence of between bilateral limbic connections. The parahippocampal gyrus, owing to its close connections with the hippocampus, has also been found to play an important role in memory processing (van Strien et al., 2009). Subsequent researches have revealed that it was also connected to both dorsal and ventral cortical regions and participates in the functional activation of default mode networks, thus reconceptualizing its multifaceted functional contributions in recent years (Aminoff et al., 2013; Seoane et al., 2022). It is believed that the parahippocampal gyrus served as a crucial bridge for the communication between “where” and “what” information streams.

The bilateral medial parahippocampal gyrus has been identified as a critical hub facilitating connectivity between the hippocampus and the posterior cingulate, parietal, and occipital cortices. This aligned with the first subtype of the parahippocampal connectome. The presence of spatial perception cells in this part that respond to spatial scene information, delivered by the parietal lobe, temporo-parieto-occipital junction area, and posterior cingulate gyrus, can provide humans with a portion of situational memory by correlating objects, people, or rewards with their location in a visual scene, providing scene information to the hippocampal memory system (Sulpizio et al., 2020; Tsitsiklis et al., 2020; Rolls and Wirth, 2018; Epstein and Baker, 2019).

On the other hand, the lateral parahippocampal (TF) gyrus exhibits prominent connections with the anterior temporal lobe, inferior temporal cortex, and hippocampus, consistent with the second subtype of the parahippocampal connectome, and is a part of the ventral information flow of the hippocampal memory system, especially in the left hemisphere, which is mainly responsible for the establishment of situational memory to communicate with the semantic information, plays an important role in the extraction of the event-related semantic information through the memory of the special events in the past (Rolls, 2018; Bonner and Price, 2013; Rolls, 2022). Interestingly, this connectome configuration also encompasses the ventral occipital region, suggesting its involvement in transmitting ventral visual information (Grill-Spector et al., 2017).

### Limitations

This study had some limitations. First, the sample size included in this study was relatively small compared to that of the HCP, and the participants only consisted of young and middle-aged individuals. Increasing the sample size and including participants from a wider age range would undoubtedly yield more accurate and objective statistical results, which can be further explored in future studies.

Second, we acknowledge that the omission of the cerebellar and brainstem regions in this study is a subject of ongoing debate. Moreover, owing to the substantial computational requirements, our study limited itself to solely employing the AAL2 parcellation scheme in conjunction with qa values to generate the brain connectivity matrix. As computational power continues to advance, future investigations should incorporate more intricate parcellation techniques, such as the HCP template, and integrate a broader range of metrics that effectively capture the complexities of brain connectivity, thereby facilitating the attainment of more comprehensive and nuanced findings.

Finally, constrained by limited funding, we were unable to procure data using a novel 7.0 T MRI scanner and instead had to make do with the highest available configuration of a 3.0 T machine. Nonetheless, considering the extensive utilization of 3.0 T MRI scanners in clinical domains, our study retains significant relevance for practical clinical applications.

## Conclusion

In the present study, we integrated 3D T1 structural MRI with diffusion MRI for a sample of 100 Chinese adults and used the Q-Space Diffeomorphic Reconstruction (QSDR) method to track white matter fibers for the ROIs within the MTL. Graph-theoretical analyses were conducted to evaluate the network topological parameters of the local structural connectomes for each ROI. Our findings demonstrated that the overall network features exhibited a low characteristic path length paired with moderate to high global efficiency, suggesting the effectiveness of the local brain connectome construction. Moreover, the amygdala connectomes exhibited longer characteristic path length and weaker global efficiency than the ipsilateral hippocampus and parahippocampal connectomes.

Additionally, we delineated the hubs within these specific connectomes: the hubs of the amygdala connectomes were dispersed across the ventral frontal, olfactory area, limbic, parietal regions and subcortical nuclei. And in the hippocampal connectomes, the hubs were mainly situated within the limbic, parietal, and subcortical regions. The hubs distribution of the parahippocampal connectomes resembled the hippocampal structural connectomes, but lacking interhemispheric connections and connectivity with subcortical nuclei.

Furthermore, we conducted hierarchical clustering for the brain local structural connectomes for each ROI. The subtypes of the bilateral amygdala connectomes were as follows: (1) The amygdala-prefrontal connectome; (2) The amygdala-ipsilateral or contralateral limbic connectome; and (3) The amygdala-posterior connectome. These connections were responsible for modulating emotions in response to threat or reward stimuli. The subtypes of the bilateral hippocampal connectomes primarily included (1) The hippocampus-ipsilateral or contralateral limbic connectome; and (2) The anterior temporal-hippocampus-ventral temporal-occipital connectome in the domain hemisphere. These subtypes were elucidated as the primary pathways corresponding to the “where” and “what” information stream. The subtypes of the parahippocampal connectomes exhibited resemblances to those of the hippocampus, and they were believed to serve as a crucial conduit for communication between the “where” and “what” information streams.

## Data Availability

The original contributions presented in the study are included in the article/supplementary material, further inquiries can be directed to the corresponding author.
